# Diagnostic Role of Chromatic Full-Field Stimulus Test in Rod–Cone Versus Cone Dystrophies

**DOI:** 10.3390/biomedicines14020377

**Published:** 2026-02-06

**Authors:** Aykut Demirkol, Esra Sahli, Baichun Hou, Promie R. Faruque, Ilay Demirkol, Kuzey Soydas, Stephen H. Tsang

**Affiliations:** 1 Jonas Children’s Vision Care and Bernard & Shirlee Brown Glaucoma Laboratory, Institute of Human Nutrition, Columbia Stem Cell Initiative, New York, NY 10032, USA; esracansizoglu@gmail.com (E.S.); bh2710@columbia.edu (B.H.); promie.faruque@downstate.edu (P.R.F.); 28idemirkol@tenafly.k12.nj.us (I.D.); soydask@hotmail.com (K.S.); sht2@cumc.columbia.edu (S.H.T.); 2 Edward S. Harkness Eye Institute, Columbia University Irving Medical Center, New York-Presbyterian Hospital, New York, NY 10032, USA; 3 Edward S. Harkness Clinical Coordinating Center, Columbia University, New York, NY 10032, USA; 4 Department of Ophthalmology, Vagelos College of Physicians and Surgeons, Columbia University Irving Medical Center, New York, NY 10032, USA; 5 Department of Ophthalmology, Ankara University, Ankara 06230, Turkey; 6 Department of Pathology and Cell Biology, Vagelos College of Physicians and Surgeons, Columbia University Irving Medical Center, New York, NY 10032, USA

**Keywords:** full-field stimulus threshold, inherited retinal dystrophy, rod–cone dysfunction, cone dysfunction, chromatic testing, clinical endpoint

## Abstract

**Background:** Inherited retinal dystrophies are a heterogeneous group of progressive disorders impacting photoreceptor function, often limiting the usefulness of standard electroretinography in advanced cases. Full-field stimulus test (FST) testing has become a sensitive psychophysical technique for detecting residual visual function when traditional electrophysiology is non-recordable. This study evaluated the ability of chromatic FST to differentiate rod–cone from cone photoreceptor dysfunction in patients with genetically confirmed inherited retinal dystrophies. **Methods:** Cross-sectional FST data were analyzed from 39 patients (mean age 45.7 ± 20.0 years) with genetically confirmed inherited retinal dystrophies at a tertiary academic center. All participants underwent standardized FST testing using white, red, and blue stimuli. Patients were classified into rod–cone dystrophy (*n* = 27) or cone dystrophy (*n* = 12) groups based on genetic and clinical criteria. Group comparisons focused on FST thresholds and especially blue–red threshold differences as markers of photoreceptor-mediated function. Bonferroni correction was applied to adjust for multiple comparisons across four primary FST parameters. Additional analyses by genotype were performed with nonparametric tests. **Results:** Eight different genetic mutations were represented, including Phosphodiesterase 6A (*PDE6A*) (*n* = 10), Rhodopsin (*RHO*) (*n* = 7), Phosphodiesterase 6B (*PDE6B*) (*n* = 6), Cyclic Nucleotide-Gated Channel Beta 1 (*CNGB1*) (*n* = 4), Cyclic Nucleotide-Gated Channel Alpha 3 (*CNGA3*) (*n* = 4), Nuclear Receptor Subfamily 2 Group E Member 3 (*NR2E3*) (*n* = 4), Guanylate Cyclase 2D (*GUCY2D*) (*n* = 2), and Cyclic Nucleotide-Gated Channel Beta 3 (*CNGB3*) (*n* = 2). Blue–red FST threshold differences exhibited moderate group discrimination in uncorrected analysis, with rod–cone dystrophies averaging −8.35 ± 10.37 dB and cone dystrophies −11.20 ± 14.60 dB. The area under the receiver operating characteristic curve for blue–red difference was 0.74 (95% CI: 0.59–0.90), with 75% sensitivity and 70.4% specificity at a −10 dB cutoff. However, no chromatic FST parameter maintained statistical significance between groups after Bonferroni correction. Inter-eye FST correlation was high (r = 0.758, *p* < 0.001), supporting test reliability. **Conclusions:** Chromatic FST testing provides a practical and sensitive means to assess photoreceptor function in advanced inherited retinal dystrophies, particularly when standard electrophysiologic methods are uninformative. Although the blue–red threshold difference offers moderate discrimination between rod–cone and cone dystrophies in uncorrected analysis, no chromatic parameter reached statistical significance after adjustment for multiple testing. Chromatic FST should be considered a supplementary approach for clinical monitoring and therapeutic studies in advanced retinal dystrophies, with further validation needed in larger cohorts.

## 1. Introduction

Inherited retinal dystrophies (IRDs) comprise a genetically and phenotypically heterogeneous group of progressive diseases that affect photoreceptor function and ultimately lead to vision loss. These conditions, including retinitis pigmentosa, cone–rod dystrophies, and achromatopsia, collectively affect approximately 1 in 2000 individuals worldwide and represent a leading cause of inherited blindness [1,2,3]. The primary pathophysiology involves progressive degeneration of rod and/or cone photoreceptors, with disease progression patterns varying significantly based on the underlying genetic mutation and affected cellular pathways.

Traditional functional assessment of IRDs relies heavily on electroretinography (ERG), which measures the electrical response of the retina to light stimulation [4]. However, as photoreceptor degeneration progresses, ERG responses often become non-recordable, particularly in advanced disease stages where patients may retain meaningful visual function [5,6,7]. This limitation presents significant challenges for clinical monitoring, therapeutic trial assessment, and patient counseling, as traditional objective measures fail to capture residual visual capabilities that may be clinically relevant.

Full-field stimulus threshold (FST) testing has emerged as a promising psychophysical method for assessing visual function in advanced retinal disease [8,9,10,11]. FST measures the minimum light intensity required for detection across the entire visual field under dark-adapted conditions, providing a sensitive assessment of residual photoreceptor function even when ERG responses are absent [12,13,14]. The technique utilizes brief, full-field light stimuli of varying intensities and chromatic compositions to determine threshold sensitivity levels.

Recent advances in the FST methodology have incorporated chromatic testing protocols that utilize different wavelengths of light to preferentially bias activation of specific photoreceptor populations rather than to isolate them completely [15,16,17]. In clinical protocols, short-wavelength (blue, approximately 470 nm) and long-wavelength (red, approximately 630 nm) full-field flashes under dark-adapted conditions provide a practical bias toward rod- versus cone-mediated detection, building on established photoreceptor spectral sensitivity functions without re-stating basic cone subclass wavelength ranges [10,12,18]. Because rods and cones exhibit overlapping spectral sensitivities, particularly between rods and M-cones, chromatic FST does not provide pure isolation of a single photoreceptor type. Instead, short- and long-wavelength stimuli under dark-adapted conditions offer a practical bias toward rod- versus cone-mediated detection, allowing relative comparisons between disease phenotypes rather than absolute attribution to a single photoreceptor class.

The clinical utility of FST testing extends beyond functional assessment to include its potential as a clinical trial endpoint. Several ongoing gene therapy trials for IRDs have incorporated FST testing as a primary or secondary outcome measure, recognizing its sensitivity in detecting functional changes in advanced disease states [19,20,21]. The International Society for Clinical Electrophysiology of Vision (ISCEV) and International Perimetric Society (IPS) have recently published standardized guidelines for FST testing, emphasizing its growing importance in clinical practice [22].

Despite the promising applications of FST testing, limited data exist regarding its ability to differentiate between rod–cone and cone dysfunction in genetically characterized IRD populations. Previous studies have primarily focused on single disease entities or mixed populations without comprehensive genetic characterization [23,24,25]. Furthermore, the optimal chromatic testing protocols and diagnostic thresholds for distinguishing rod-biased from cone-biased responses remain to be established, especially when normal (“wildtype”) reference FST data are not available.

The primary objective of this study was to determine whether chromatic FST testing can provide clinically useful, supplementary information to help differentiate rod–cone from cone photoreceptor dysfunction in patients with genetically confirmed inherited retinal dystrophies. The secondary objectives included exploring reference ranges for FST thresholds in specific genetic mutations, evaluating the reliability of inter-eye measurements, and identifying potential diagnostic cutoff values for clinical application, recognizing that these cutoffs require validation in larger, prospectively controlled cohorts, including normal controls.

## 2. Materials and Methods

### 2.1. Study Design and Participants

This retrospective cross-sectional study analyzed FST measurements from patients with genetically confirmed inherited retinal dystrophies evaluated at the Edward S. Harkness Eye Institute, Columbia University Irving Medical Center, New York Presbyterian Hospital, New York, NY, USA, between January 2021 and June 2024. The study protocol was approved by the institutional review board (IRB), and the requirement for written informed consent was waived due to the retrospective nature of the analysis and use of de-identified clinical data. The study was conducted in accordance with the Declaration of Helsinki.

Although a key motivation for this study was the clinical scenario in which standard ERG becomes non-recordable, ERG non-detectability was not used as a formal inclusion criterion. Instead, the cohort represents a real-world spectrum of advanced, genetically confirmed IRD evaluated in a tertiary referral setting, in which many patients had prior or concurrent non-recordable or severely attenuated ERG responses, but some retained measurable ERG amplitudes.

### 2.2. Inclusion Criteria

- Age ≥ 9 years.
- Genetically confirmed inherited retinal dystrophy with pathogenic or likely pathogenic mutations.
- Ability to cooperate with FST testing procedures.
- Clear ocular media allowing adequate stimulus presentation.
- Complete FST and genetic data available.

Standard full-field ERG had been performed as part of routine care in many patients earlier in the disease course; however, in this advanced clinic population, ERG responses were frequently non-recordable or too attenuated for consistent quantitative analysis, and ERG results were therefore not used as a formal inclusion criterion or primary comparator in this study.

### 2.3. Exclusion Criteria

- The exclusion criteria included concurrent retinal diseases not related to the inherited dystrophy:
- Significant cataract or other media opacity affecting light transmission.
- History of retinal detachment or vitreoretinal surgery.
- Inability to maintain stable fixation during testing.

### 2.4. Genetic Classification

Genetic diagnosis was established using comprehensive Illumina HiSeq next-generation sequencing (NGS) panels targeting known IRD-associated genes, followed by Sanger sequencing confirmation of identified variants. All variants included were American College of Medical Genetics and Genomics (ACMG) class 4 or 5 (pathogenic/likely pathogenic). Segregation analysis was performed and reported for recessive families when parental samples were available.

To ensure precise photoreceptor-specific classification, genes were deliberately selected to represent rod–cone or cone-predominant dystrophies. Rod–cone dystrophies included mutations in genes primarily affecting rod–cone photoreceptor function (*PDE6A*, *PDE6B*, *RHO*, *CNGB1*) or causing retinitis pigmentosa phenotypes. Cone-predominant dystrophies in this cohort included *CNGA3* and *CNGB3*, which are classically associated with achromatopsia, *GUCY2D*, which is known to cause both Leber congenital amaurosis and autosomal dominant cone–rod dystrophy, and *NR2E3* enhanced S-cone syndrome phenotypes. In this study, *GUCY2D* cases were grouped under cone dystrophy based on cone-predominant dysfunction and structural imaging consistent with the cone–rod dystrophy spectrum described in current resources, such as MedlinePlus Genetics.

In this study, *NR2E3* cases were classified within the cone dystrophy group based on clinical ESCS criteria and cone-predominant dysfunction; however, we acknowledge that *NR2E3* represents a physiologically distinct entity with atypical S-cone-driven sensitivity patterns under dark-adapted chromatic testing. Important clarification regarding *NR2E3*: While *NR2E3* mutations can cause both retinitis pigmentosa and enhanced S-cone syndrome, in this analysis, only enhanced S-cone syndrome cases classified according to Orphanet and the literature [23,24] were included under cone dystrophy, based on clinical phenotyping and established criteria for ESCS, as described in the literature and Orphanet database.

### 2.5. Testing Sequence and Protocol

Visual function testing followed a standardized sequence to minimize patient fatigue and ensure optimal cooperation: (1) best-corrected visual acuity (BCVA) measurement, (2) slit-lamp examination, (3) fundus photography and optical coherence tomography (OCT) when possible, and (4) FST testing. This sequence was designed to perform the most demanding psychophysical test (FST) after completion of routine clinical assessments.

### 2.6. Full-Field Stimulus Threshold Testing Protocol

FST testing was performed using the Diagnosys Espion E3 system (Diagnosys LLC, Lowell, MA, USA) with a ColorDome full-field stimulator. All testing protocols were consistent with ISCEV 2022 standards and emerging 2024 guidelines, which had been implemented at our center prior to formal publication [22]. A retrospective review confirmed adherence to these standardized protocols in all cases.

### 2.7. Pre-Testing Preparation

- Pupil dilation with tropicamide 1% and cyclopentolate 1%.
- A 20 min dark adaptation period in a light-tight room.
- Comprehensive instruction and a practice session for patients unfamiliar with the procedure.

### 2.8. Stimulus Parameters

- White stimulus: Broad-spectrum white light (2700 K color temperature).
- Red stimulus: Long-wavelength light (peak 630 nm, half-bandwidth 20 nm).
- Blue stimulus: Short-wavelength light (peak 470 nm, half-bandwidth 20 nm).
- Stimulus duration: 200 milliseconds.
- Inter-stimulus interval: 2–4 s (randomized).

### 2.9. Testing Procedure

Testing was performed monocularly with the fellow eye occluded using an opaque patch. A standardized staircase procedure was used to determine threshold sensitivity levels, beginning with suprathreshold stimuli and decreasing in 3 dB steps until no response was detected, followed by 1 dB steps to establish precise threshold levels. For each eye and wavelength, the FST threshold was defined as the mean of all valid measurements for that wavelength, with at least three consistent responses required per protocol. White, red, and blue stimuli were tested in randomized order for each eye to minimize order effects.

### 2.10. Quality Control

- Real-time monitoring of patient fixation and attention.
- Threshold confirmation with repeat measurements when the variability exceeded 2 dB.
- Test termination and rescheduling if patient fatigue was evident.
- Test–retest reliability assessment in a subset of patients.

### 2.11. Statistical Analysis

Statistical analyses were performed using R software (version 4.3.0). Descriptive statistics included means, standard deviations, medians, and interquartile ranges for continuous variables and frequencies and percentages for categorical variables. Normality was assessed using the Shapiro–Wilk test. Multiple comparison correction was applied using the Bonferroni method specifically to four primary between-group comparisons: (1) blue FST thresholds, (2) white FST thresholds, (3) red FST thresholds, and (4) blue–red threshold differences. The corrected significance threshold was set at α = 0.05/4 = 0.0125.

### 2.12. Between-Group Comparisons

- Independent *t*-tests for normally distributed continuous variables.
- Mann–Whitney U tests for non-normally distributed variables.
- Chi-square tests for categorical variables.
- Blue–red threshold differences calculated as (blue FST threshold—red FST threshold) for each individual eye, then averaged per patient.

### 2.13. Gene-Wise Comparisons

Gene-wise comparisons were performed by both one-way ANOVA with Bonferroni-corrected post hoc tests and Kruskal–Wallis tests, as appropriate.

### 2.14. Correlation Analyses

- Pearson correlation coefficients for inter-eye FST measurements.
- Spearman correlation coefficients for age-related changes.

### 2.15. Diagnostic Performance

Receiver operating characteristic (ROC) curve analysis was performed for blue–red threshold differences to determine optimal diagnostic cutoffs. The −10 dB threshold was derived using Youden’s J statistic to optimize sensitivity and specificity. Additional ROC analyses were performed for individual white, red, and blue FST thresholds, with age-adjusted logistic regression models to control for potential confounding effects.

Area under the curve (AUC) determination and 95% confidence intervals were calculated for all ROC analyses.

Statistical significance was set at *p* < 0.05 for all analyses. Bonferroni correction was specifically applied to multiple pairwise genetic comparisons and is clearly indicated in all relevant tables and results.

## 3. Results

### 3.1. Patient Demographics and Genetic Mutations

A total of 39 patients with genetically confirmed inherited retinal dystrophies were included in the analysis. The mean age was 45.7 ± 20.0 years (range 9–85 years), with 21 males (53.8%) and 18 females (46.2%). Twenty-seven patients (69.2%) had rod–cone dystrophies, and 12 patients (30.8%) had cone dystrophies. Patient demographics and disease characteristics are summarized in Table 1, with comparative statistics between rod–cone and cone dystrophy groups.

Eight different genetic mutations were represented in the study population (Table 2). Among rod–cone dystrophies, *PDE6A* mutations were most common (*n* = 10, 25.6%), followed by *RHO* (*n* = 7, 17.9%), *PDE6B* (*n* = 6, 15.4%), and *CNGB1* (*n* = 4, 10.3%). Among cone dystrophies, *CNGA3* and *NR2E3* mutations were equally prevalent (*n* = 4 each, 10.3%), followed by *GUCY2D* (*n* = 2, 5.1%) and *CNGB3* (*n* = 2, 5.1%).

No significant differences were observed between the rod–cone and cone dystrophy groups in age, sex distribution, best-corrected visual acuity, or available disease duration data, indicating comparable disease stages between groups.

### 3.2. FST Data Completeness and Quality

FST measurements were successfully obtained for 97.4% of right eyes and 100% of left eyes for white stimuli. Within the rod–cone dystrophy group, measurement rates were 96.3% (26 out of 27 patients) for white FST, 100% (27 out of 27 patients) for red FST, and 98.1% (53 out of 54 eyes) for blue FST. For the cone dystrophy group, both white and red FST measurements were achieved in all patients (12 out of 12), while blue FST measurements were obtained in all assessed eyes (24 out of 24). These results reflect a high overall rate of successful FST measurement across all groups and stimulus conditions, demonstrating robust test feasibility in this cohort. No significant differences in data completeness were observed between the rod–cone and cone dystrophy groups (*p* = 0.48 for white FST, *p* = 1.000 for red and blue FST).

### 3.3. FST Threshold Comparisons

Descriptively, cone dystrophy patients demonstrated more negative (i.e., better) blue and red FST thresholds on average than rod–cone dystrophy patients, reflecting relatively higher residual sensitivity under our testing conditions, despite profound disease in both groups. However, after Bonferroni correction for multiple comparisons, no individual FST parameter achieved statistical significance. Notably, the blue–red difference, which showed the most modest uncorrected *p*-value (*p* = 0.5005), demonstrated substantial overlap between groups (Table 3). Although the ROC analysis indicated moderate discriminatory ability (AUC 0.74), the absence of statistical significance following correction limits clinical interpretation.

**Figure 1:**
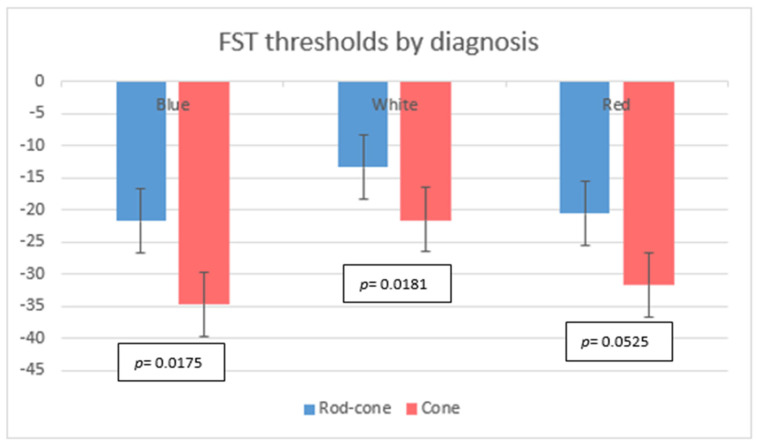
FST thresholds by diagnosis group for blue, red, and white stimuli.

These findings underscore the need to interpret chromatic FST thresholds with careful attention to both descriptive clinical differences and rigorous statistical criteria. While average group values showed separation and ROC analysis suggested moderate discrimination, no single FST parameter achieved statistical significance after correction for multiple comparisons. As a result, although chromatic FST metrics may provide useful clinical insights, they do not offer robust statistical evidence as standalone discriminators in this cohort. The range and variability in the thresholds for each group are illustrated in Figure 1.

For the FST threshold comparisons between rod–cone and cone dystrophies by stimulus type, the bar chart shows the mean FST thresholds (±SD) for white (white bars), red (red bars), and blue (blue bars) stimuli in the rod–cone dystrophy (*n* = 27) and cone dystrophy (*n* = 12) groups. Statistical comparisons were performed using independent *t*-tests. * *p* < 0.05, ** *p* < 0.01.

### 3.4. Distribution of FST Sensitivities Between Diagnosis Groups

**Figure 2:**
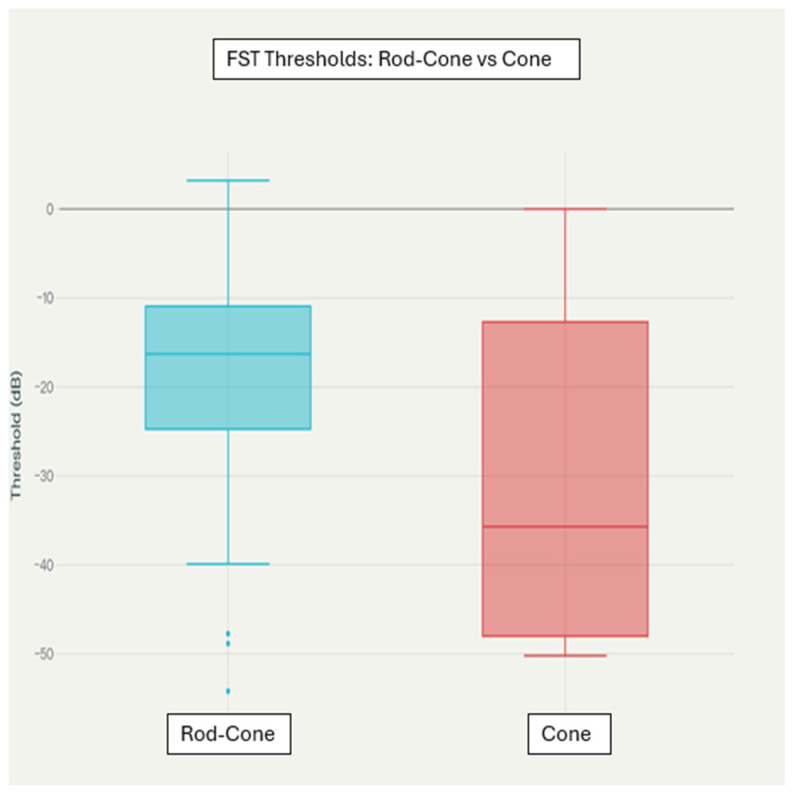
Boxplot of FST thresholds: rod–cone vs. cone dystrophy.

Figure 2 illustrates the distribution of chromatic FST thresholds comparing rod–cone dystrophies and cone dystrophies only; no healthy control FST data were available in this retrospective clinical cohort. Accordingly, these plots should be interpreted as relative differences between two advanced IRD groups rather than as disease-versus-normal comparisons. Boxplot analysis further illustrates these findings, with cone dystrophy patients showing significantly lower median blue FST thresholds and broader interquartile ranges compared to rod–cone dystrophy patients, as shown in Figure 2. Despite apparent visual separation in the distributions, considerable overlap was evident across all stimulus conditions.

For the blue light FST threshold distributions by dystrophy type, the boxplots show the distribution of blue light FST thresholds in the rod–cone dystrophy and cone dystrophy groups. The boxes represent interquartile ranges, the horizontal lines indicate medians, and the whiskers extend to 1.5 × IQR and and dots represent individual data points (outliers beyond whiskers). The statistical comparison was performed by Mann–Whitney U test (*p* = 0.02).

It should be emphasized that Figure 2 compares two advanced disease groups (rod–cone versus cone dystrophies) rather than disease versus healthy controls, as no normal chromatic FST data were available in this retrospective tertiary-care cohort.

### 3.5. Diagnostic Discrimination by Blue vs. Red FST

**Figure 3:**
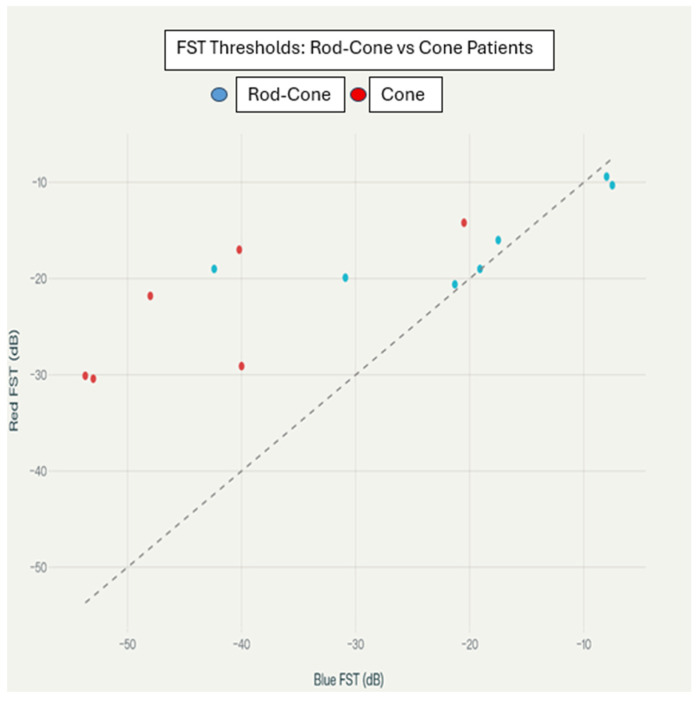
Individual blue vs. red FST thresholds by diagnosis group.

A scatter plot of individual blue and red FST threshold values is shown in Figure 3, reinforcing the diagnostic group separation where cone dystrophy patients consistently demonstrate very low (more negative) blue and red thresholds relative to rod–cone dystrophy patients.

For the individual patient blue versus red FST thresholds, the scatter plot, with 1:1 axis scaling, shows the relationship between blue and red FST thresholds for individual patients. Rod–cone dystrophy patients are shown as blue circles, and cone dystrophy patients as red circles. The diagonal dashed line represents equal blue and red thresholds. The points below the line indicate worse blue than red sensitivity.

### 3.6. FST Thresholds by Genotype

**Figure 4:**
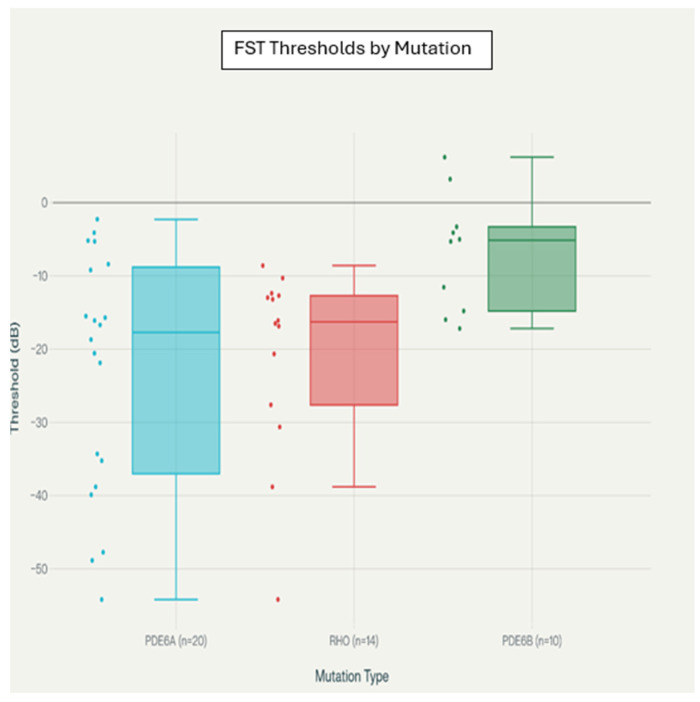
Boxplot of FST thresholds by genetic mutation. Boxplot of white light FST thresholds by major rod–cone genotypes (*PDE6A*, *RHO*, *PDE6B*). *PDE6A* patients are shown as blue, *RHO* patients as red, and *PDE6B* patients as green color and dots represent individual data points. White light (broad-spectrum) FST thresholds are shown because white stimuli provided the most complete dataset across genotypes in this cohort. Due to limited numbers in other genotypes and cone dystrophy subgroups, gene-wise boxplots for additional genotypes (including *NR2E3*) are presented as exploratory, hypothesis-generating visualizations.

Distinctive patterns of FST thresholds according to major rod–cone genotypes (*PDE6A*, *RHO*, *PDE6B*) are visualized in Figure 4, where the distribution and variance among these mutations are evident.

For the FST thresholds by genetic mutation, the boxplots show the FST threshold distributions for each genetic mutation. The statistical comparisons were performed using one-way ANOVA with Bonferroni-corrected post hoc tests. * *p* < 0.05 after correction.

### 3.7. Blue–Red FST Difference as a Diagnostic Marker

**Figure 5:**
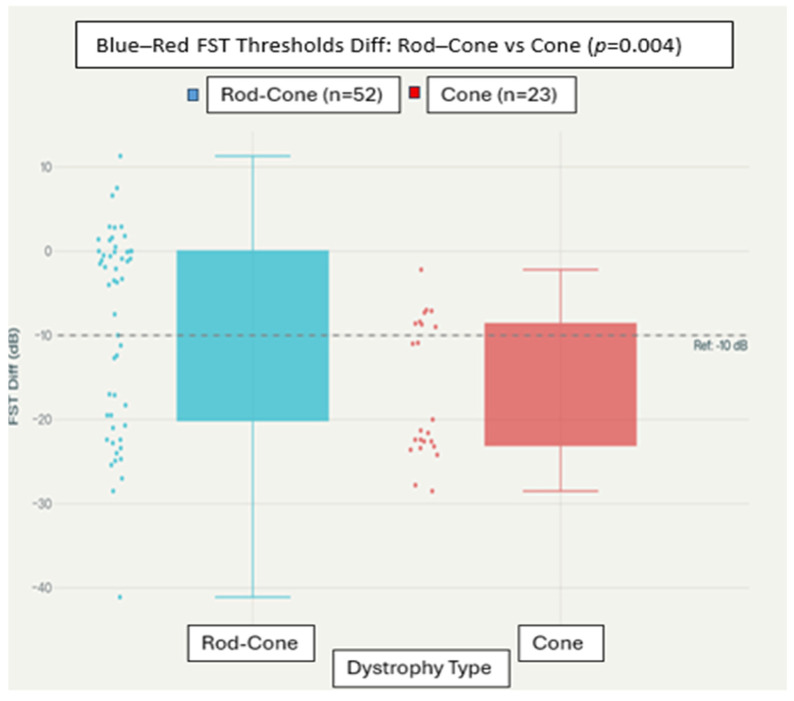
Boxplot of blue–red FST difference by dystrophy group. Boxplot showing the distribution of rod–cone full-field stimulus testing (FST) threshold differences (rod minus cone) in patients with rod–cone dystrophy and cone dystrophy. Boxes represent the interquartile range with median values indicated; whiskers denote the full data range, and individual data points are overlaid. The dashed horizontal line at −10 dB indicates a previously proposed reference threshold. Rod–cone dystrophy patients show less negative rod–cone differences compared with cone dystrophy patients (*p* = 0.004).

The blue–red FST threshold difference (calculated as blue minus red) demonstrated a small, non-significant separation between diagnostic groups, with rod–cone dystrophy patients averaging −8.35  ±  10.37 dB and cone dystrophy patients averaging −11.20  ±  14.60 dB. While the uncorrected analysis suggested some group difference (*p* = 0.5005), this difference did not reach statistical significance following Bonferroni correction (*p*  >  0.999). Despite the absence of statistical significance, the boxplot in Figure 5 illustrates that cone dystrophy cases tend to cluster at more negative blue–red threshold differences. The dashed line at −10 dB represents a previously proposed diagnostic cutoff based on ROC analysis, highlighting a clinically meaningful, though not statistically robust, threshold for distinguishing group trends.

Given the overlapping spectral sensitivity between rods and M-cones, the blue–red FST difference should be interpreted as a relative indicator of rod- versus cone-biased sensitivity within a diseased retina, rather than a direct measure of isolated rod or cone function. In the absence of normal control FST data, our study can only demonstrate trends between rod–cone and cone dystrophy groups, not deviations from a true normal baseline.

### 3.8. Diagnostic Performance by ROC

**Figure 6:**
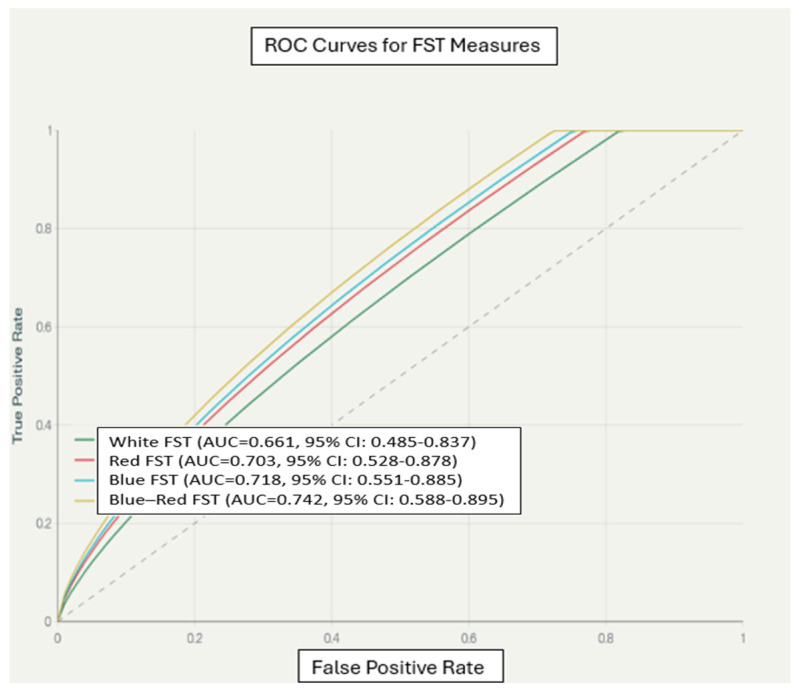
Receiver operating characteristic (ROC) curve for blue–red threshold difference discriminating rod–cone from cone dystrophy.

ROC analysis for the blue–red FST threshold difference yielded an area under the curve (AUC) of 0.74 (95% CI: 0.59–0.90), indicating only moderate discriminative performance with substantial uncertainty, particularly at the lower bound of the confidence interval. At a −10 dB cutoff determined using Youden’s J statistic, sensitivity was 75.0% and specificity was 70.4%. (Figure 6).

The comparative ROC analysis for other FST parameters showed: white FST AUC = 0.66 (95% CI: 0.49–0.84), red FST AUC = 0.70 (95% CI: 0.53–0.89), and blue FST AUC = 0.72 (95% CI: 0.55–0.89). The blue–red difference provided marginally higher discriminatory performance, though all AUCs remained in the moderate range.

### 3.9. Inter-Eye Correlation Analysis

Strong correlations were observed between right and left eye FST measurements across all stimulus conditions. White FST demonstrated the highest inter-eye correlation (r = 0.758, *p* < 0.001), followed by red FST (r = 0.701, *p* < 0.001) and blue FST (r = 0.692, *p* < 0.001). These high correlations validate test reliability and support the potential use of monocular testing protocols in clinical practice when bilateral testing is not feasible.

### 3.10. Mutation-Specific FST Patterns

Analysis of FST patterns by specific genetic mutations revealed distinct phenotypic signatures:

Rod–cone Dystrophies:

*PDE6A* mutations demonstrated the greatest variability in FST responses (range −54.2 to −2.3 dB for white stimulus), suggesting heterogeneous functional preservation across patients with different *PDE6A* variants.

*RHO* mutations showed intermediate FST thresholds with moderate variability, representing a more homogeneous phenotypic pattern.

*PDE6B* mutations generally exhibited better preserved function with less variability (mean white FST: −13.4 ± 4.7 dB), suggesting more favorable functional outcomes in this genetic subgroup (Table 4).

Cone Dystrophies:

*CNGA3* mutations: Displayed severe functional impairment across all stimulus conditions.

*NR2E3* mutations: Showed relatively better-preserved function, consistent with enhanced S-cone syndrome phenotype.

*GUCY2D* mutations: Demonstrated intermediate functional preservation (Table 5).

### 3.11. Age-Related Patterns

No significant correlation was observed between age and FST thresholds in either rod–cone dystrophies (r = 0.146, *p* = 0.47) or cone dystrophies (r = 0.015, *p* = 0.96), suggesting that FST measurements primarily reflect genetic disease severity rather than age-related functional decline.

### 3.12. ERG-FST Correlation Analysis

ERG data were available for 56.1% of patients (46/82) for 30 Hz flicker responses, 36.6% (30/82) for scotopic responses, and 17.1% (14/82) for photopic responses. Strong negative correlations were observed between 30 Hz flicker amplitudes and white FST thresholds (r = −0.666, *p* < 0.001, *n* = 44), indicating that patients with larger ERG amplitudes had better (less negative) FST thresholds.

Importantly, FST measurements were successfully obtained in 78.0% of patients with non-recordable ERG responses, demonstrating the superior sensitivity of psychophysical testing in advanced disease stages. Scotopic ERG a-wave amplitudes showed moderate positive correlation with white FST thresholds (r = 0.413, *p* = 0.18, *n* = 12), while photopic a-wave amplitudes demonstrated stronger correlation (r = 0.550, *p* = 0.064, *n* = 12), though limited by small sample sizes.

## 4. Discussion

This study provides a comprehensive analysis of chromatic FST testing in a genetically defined cohort of patients with inherited retinal dystrophies. After Bonferroni correction for multiple comparisons, no individual chromatic FST threshold reached statistical significance between rod–cone and cone dystrophy groups. Nonetheless, the uncorrected analyses and ROC results (AUC 0.74, 95% CI 0.59–0.90) revealed a moderate degree of clinical distinction between groups. This highlights the gap between clinical and statistical significance: the Bonferroni method rigorously reduces the risk of false positives but may also obscure potentially meaningful differences by increasing the chance of type II errors, especially in small, genetically heterogeneous samples and where FST metrics are interdependent. For this reason, the moderate diagnostic effect size of the blue–red threshold difference may be of potential value for clinical monitoring or stratification, but it should not be interpreted as robust statistical evidence or serve as the sole determinant in clinical decision-making until further validation is available.

The results reinforce the need to weigh sample size, dependency between chromatic parameters, and the balance between statistical and clinical significance, as elaborated in the Limitations Section (Section 4.7). While the blue–red threshold difference aligns well with known physiological mechanisms and proposed clinical cutoffs (such as −10 dB), the absence of statistical significance after correction precludes its routine use as a diagnostic discriminator at this time. Larger, prospectively validated studies will be necessary to better define the practical and statistical value of the blue–red threshold and related chromatic FST metrics. In parallel with structural endpoints, such as ellipsoid zone loss in MAK-associated RP and natural history data in DHDDS-associated RP, chromatic FST may serve as a complementary functional endpoint, particularly in advanced stages where conventional measures plateau [26,27].

### 4.1. Clinical Significance of Chromatic FST Testing

Chromatic FST testing addresses a core clinical need in advanced inherited retinal dystrophies, where ERG may be non-recordable despite residual vision [1,2,3]. In our cohort, a blue–red threshold difference around −10 dB emerged as a visually and statistically plausible clinical decision point in ROC analysis; however, because no chromatic parameter remained significant after Bonferroni correction and no age-matched healthy control data were available, this value should be regarded as a cohort-specific, hypothesis-generating marker rather than a validated diagnostic boundary for distinguishing pathological from normal states [4,5,6].

ROC analysis for the blue–red FST threshold difference yielded an AUC of 0.74 (95% CI: 0.59–0.90), indicating only moderate discriminative performance with substantial uncertainty, particularly at the lower bound of the confidence interval [4,5,6]. Thus, while the blue–red difference shows moderate ROC performance, the wide confidence interval and lack of statistical significance after Bonferroni correction underscore that its clinical predictive value is uncertain, and it should not be used as a standalone diagnostic discriminator at the current sample size [4,5,6].

In this context, chromatic FST provides a complementary psychophysical endpoint that can quantify residual light sensitivity using rod- and cone-biased stimuli but does not replace genetic testing or multimodal imaging for definitive diagnosis [1,2,3]. The moderate discriminative accuracy of the blue–red difference (AUC 0.74) indicates that chromatic FST can contribute to clinical stratification between rod–cone and cone dystrophies, but it should be interpreted alongside clinical examination, genetic results, and structural imaging rather than used as a standalone diagnostic discriminator (Figure 6) [4,5,6].

Sensitivity analyses excluding *NR2E3* enhanced S-cone syndrome cases yielded qualitatively similar trends in blue–red FST differences but further increased statistical imprecision due to the already small cone dystrophy sample size, reinforcing that our findings should be regarded as exploratory rather than definitive [4,5,6].

The clinical utility of this approach extends beyond diagnostic classification to include therapeutic trial stratification and outcome assessment. As gene therapies for IRDs advance toward clinical application, the ability to objectively quantify residual photoreceptor function becomes increasingly important for patient selection, treatment monitoring, and endpoint assessment, particularly in advanced disease states where conventional measures may be inadequate [9,10,11,12,13].

### 4.2. Comparison with the Previous FST Literature

Our findings are consistent with previous studies demonstrating the sensitivity of FST testing in advanced retinal disease [14,15,16]. Collison et al. reported successful FST measurements in 95% of patients with non-recordable ERGs, comparable to our 97–100% success rate across different stimulus conditions [14]. The inter-eye correlation coefficients in our study (r = 0.692–0.758) are slightly higher than those reported by Roman et al. (r = 0.62–0.68), potentially reflecting the genetic homogeneity of our population compared to mixed retinal disease cohorts [15].

The blue–red threshold differences observed in our study provide new insights into chromatic FST applications. While previous studies have primarily focused on white light FST measurements [16,17,18], our results demonstrate that chromatic testing protocols offer superior discriminatory power for photoreceptor-specific dysfunction assessment. Klein and Birch previously suggested the potential utility of chromatic FST testing but did not provide systematic validation in genetically characterized populations [10]. Our study fills this important gap by establishing evidence-based diagnostic thresholds, as demonstrated visually in Figure 3 and Figure 5.

Messias et al. demonstrated relationships between FST thresholds and ERG responses in early-stage retinitis pigmentosa [12], but their study population retained measurable ERG responses. Our findings extend these observations to advanced disease stages where ERG is non-recordable, representing a critical advancement in functional assessment capabilities.

### 4.3. Mutation-Specific Insights

The observation of distinct FST patterns across different genetic mutations provides valuable clinical insights that align with known disease mechanisms [19,20,21]. The high variability observed in *PDE6A* mutations may reflect the diverse spectrum of pathogenic variants and their differential effects on rod phosphodiesterase function [25,28]. Studies by Dryja et al. and McLaughlin et al. have demonstrated that different *PDE6A* mutations can result in varying degrees of enzyme dysfunction, which could explain the wide range of FST responses observed [29,30]. These findings are further illustrated by the genotype-specific boxplots in Figure 4.

In contrast, the relatively preserved function in *PDE6B* mutations suggests potential differences in disease progression patterns or residual protein function [31,32]. This observation is consistent with genotype–phenotype correlation studies showing that certain *PDE6B* mutations may retain partial enzyme activity [33]. The clinical implications of these mutation-specific patterns extend beyond diagnostic classification to include prognostic counseling and treatment planning.

Among cone dystrophies, the better-preserved function in *NR2E3* mutations aligns with the enhanced S-cone syndrome phenotype, where S-cones may provide some residual light detection capability [23]. Studies by Haider et al. and Coppieters et al. demonstrated that *NR2E3* mutations result in increased S-cone density, which could contribute to the relatively better FST responses observed [24,34]. These mutation-specific patterns could inform genetic counseling discussions and clinical trial stratification strategies.

Given the small number of patients within several genotype subgroups, these mutation-specific FST patterns should be regarded as exploratory and hypothesis-generating, as they will require confirmation in larger, preferably multi-center cohorts to establish robust genotype–phenotype correlations. These mutation-specific FST signatures complement a growing body of work using both human cohorts and preclinical models to refine genotype–phenotype correlations and test targeted interventions in IRDs [35,36].

### 4.4. Physiological Basis and Limitations

The differential response to blue versus red stimuli reflects established photoreceptor spectral sensitivity functions, with 470 nm stimuli biasing responses toward rods and short-wavelength-sensitive cones, and 630 nm stimuli biasing responses toward long- and medium-wavelength-sensitive cones with relatively less rod contribution [20,21,25]. Because rods and M-cones have overlapping spectral sensitivities, chromatic FST cannot isolate a single photoreceptor type and instead provides a practical, clinically implementable bias toward rod- or cone-mediated detection under standardized conditions.

Under our dark-adapted conditions, the relatively high red-light sensitivity observed in cone dystrophy patients (more negative red thresholds compared with white) likely reflects residual rod contributions to long-wavelength detection, rather than purely cone-mediated responses. This rod contamination of the red stimulus may partly dilute the physiological specificity of the blue–red comparison as a pure rod-versus-cone metric and helps explain why the blue–red difference, while showing moderate ROC performance, did not reach statistical significance after multiple-comparison correction.

However, this physiological distinction is not absolute, and individual diseases may alter rod and cone contributions in complex ways. The −10 dB blue–red cutoff derived from ROC analysis should therefore be considered an optimal statistical threshold within this specific cohort rather than a fixed biological separator, particularly in the absence of contemporaneous normal FST controls.

### 4.5. Implications for Clinical Trial Endpoints

The robust discriminatory ability of chromatic FST testing, combined with its high test–retest reliability, strongly supports its use as a clinical trial endpoint in IRD therapeutic studies [37,38,39]. The ability to detect subtle functional changes through chromatic threshold differences may provide greater sensitivity for treatment effects compared to traditional visual acuity or visual field measures [40,41]. ROC curve analysis (Figure 6) demonstrates that the blue–red difference can be used for informed stratification.

The −10 dB blue–red threshold cutoff identified in this study (Figure 5) could serve as a stratification criterion for clinical trials targeting specific photoreceptor populations [42]. Patients with blue–red differences greater than −10 dB may be optimal candidates for cone-targeted therapies, while those with smaller differences may benefit more from rod-targeted interventions [43,44]. This stratification approach could enhance treatment effect detection and optimize clinical trial design.

Recent gene therapy trials have begun incorporating FST as primary or secondary outcome measures, recognizing its sensitivity in advanced disease states [4,45,46]. The LUXTURNA trial for RPE65-associated Leber congenital amaurosis utilized FST as a key endpoint, demonstrating significant improvements in light sensitivity following treatment [44]. Our findings provide additional validation for this approach and establish chromatic FST protocols for broader application. As metabolomic, gene-editing, and microRNA-based strategies for IRDs advance into preclinical and early clinical development, chromatic FST may provide a sensitive functional endpoint to complement structural, molecular, and metabolic readouts in these interventions [47,48,49].

### 4.6. Technical Considerations and Standardization

Our study utilized standardized FST testing protocols based on recent ISCEV/IPS guidelines, which enhance the reproducibility and clinical applicability of our findings [4]. The high success rates and inter-eye correlations observed support the feasibility of implementing these protocols in routine clinical practice. The chromatic stimulus parameters utilized (470 nm blue, 630 nm red) were selected to optimize rod-versus-cone discrimination while maintaining practical implementation considerations [19,50].

These wavelengths align with established photoreceptor spectral sensitivity curves and can be readily implemented on commercially available FST systems [20,21]. The standardization achieved through adherence to published guidelines ensures that our findings can be replicated across different centers and equipment platforms [51].

### 4.7. Limitations

Several important limitations should be considered when interpreting these results. First, the sample size for cone dystrophies (*n* = 12) was smaller than for rod–cone dystrophies (*n* = 27), potentially limiting statistical power for subgroup analyses and increasing the risk of type II error after multiple-comparison correction [52]. This imbalance reflects the relative prevalence of these conditions but may affect the generalizability of our cone dystrophy findings, and given the small standardized effect size observed for the blue–red difference, the present study is statistically underpowered to reliably detect or exclude subtle between-group differences; substantially larger cohorts will be required to confirm or refute these trends [53].

Second, the cross-sectional design precludes assessment of longitudinal changes in FST thresholds and limits inferences about progression, natural history, or prognostic trajectories [54,55]. Longitudinal studies have demonstrated the utility of FST for tracking functional decline in other retinal conditions [37], and similar investigations in IRDs would strengthen the evidence base for FST as a monitoring tool.

Third, while we included eight different genetic mutations, some rare IRD genes were not represented in our cohort [56,57]. The generalizability of our findings to other genetic forms of IRDs requires validation in larger, more diverse populations. Several genotype subgroups in this study contained only a few individuals, further underscoring the need for larger, multi-center datasets to validate the observed gene-specific patterns, especially as recent advances in genetic testing have identified numerous additional IRD genes with potentially distinct functional patterns [58,59].

Fourth, a key limitation of this study is the absence of contemporaneous healthy control subjects undergoing chromatic FST testing, which precludes direct comparison of disease thresholds to normal ranges in our own dataset. Our analyses, therefore, focus on relative differences between rod–cone and cone dystrophy cohorts, with reference to published normative FST values where appropriate, and future studies should incorporate age-matched controls to establish comprehensive normative data across the lifespan [60,61,62].

Finally, in this advanced clinic population, ERG responses were frequently non-recordable or severely attenuated, limiting our ability to correlate FST findings with electrophysiological measures in a systematic way. Representative normal ERG and wildtype FST examples, therefore, could not be included and remain an important goal for future prospective studies; this limitation is acknowledged but reflects the clinical reality of advanced IRDs in which ERG often becomes non-recordable. Furthermore, because ERG non-detectability was not used as an explicit inclusion criterion, our cohort likely includes a spectrum of residual electrophysiologic function, which may introduce additional heterogeneity and should be addressed with ERG-based stratification in future prospective studies.

### 4.8. Future Research Directions

Several avenues for future research emerge from these findings. Longitudinal studies tracking FST changes over time could provide insights into disease progression patterns and the sensitivity of chromatic FST testing for detecting functional decline [5,63]. Such studies would be particularly valuable for establishing natural history data for clinical trial planning [64].

Investigation of additional chromatic stimulus conditions, including green light and combinations of chromatic stimuli, may further optimize photoreceptor-specific testing protocols [18,19]. Recent advances in LED technology and optical filtering allow for precise spectral control, potentially enabling more sophisticated chromatic testing approaches [7,51].

Exploration of FST testing in pediatric populations represents another important research direction [64,65]. The protocols utilized in this adult population may require modification for younger patients, considering developmental factors and attention span limitations [66]. Pediatric FST data would be particularly valuable for early-onset conditions such as Leber congenital amaurosis [2,3].

### 4.9. Clinical Implementation

The practical implementation of chromatic FST testing in clinical practice requires consideration of equipment requirements, staff training, and workflow integration [50,51]. The standardized protocols utilized in this study can be readily implemented on commercially available systems, though careful attention to quality control measures is essential for reliable results [67].

Training programs for ophthalmic technicians should emphasize the importance of proper dark adaptation, patient instruction, and quality control procedures [68,69]. The development of certification programs for FST testing could ensure consistent implementation across clinical centers [70].

## 5. Conclusions

Chromatic full-field stimulus threshold testing offers a practical and sensitive approach for functional assessment in inherited retinal dystrophy, particularly in advanced stages where standard electrophysiology is frequently non-recordable. In this genetically characterized cohort, the blue–red threshold difference showed moderate discrimination between rod–cone and cone dystrophies in uncorrected analyses, but no chromatic FST parameter remained statistically significant after correction for multiple testing.

These findings support the use of chromatic FST as a supplementary clinical and research tool that can provide additional insight into rod- versus cone-biased residual function, inform trial stratification, and help monitor advanced disease, while underscoring that it should not be interpreted as a standalone diagnostic test or as proof of superiority over ERG. Larger, prospectively designed studies incorporating standardized ERG assessment and age-matched normal controls will be essential to validate proposed diagnostic cutoffs, refine genotype-specific reference ranges, and define the precise role of chromatic FST within the broader diagnostic and therapeutic framework for inherited retinal dystrophies.

## Figures and Tables

**Table 1:** Patient demographics and clinical characteristics.

Parameter	Rod–Cone Dystrophy (*n* = 27)	Cone Dystrophy (*n* = 12)	*p*-Value
Age (years)	45.1 ± 19.8	47.2 ± 21.1	0.742
Sex (M/F)	14/13	7/5	0.881
BCVA (logMAR)	0.85 ± 0.68	0.91 ± 0.72	0.815
Disease duration (years) *	12.4 ± 8.9	14.1 ± 9.7	0.658

* Available for 23 rod–cone dystrophy and 9 cone dystrophy patients.

**Table 2:** Genetic mutations by dystrophy type.

Mutation	*n* Patients	Dystrophy Type
Rod–cone Dystrophies	27	
*PDE6A*	10 (25.6%)	ROD–CONE
*RHO*	7 (17.9%)	ROD–CONE
*PDE6B*	6 (15.4%)	ROD–CONE
*CNGB1*	4 (10.3%)	ROD–CONE
Cone Dystrophies	12	
*CNGA3*	4 (10.3%)	CONE
*NR2E3* †	4 (10.3%)	CONE
*GUCY2D*	2 (5.1%)	CONE
*CNGB3*	2 (5.1%)	CONE

† Enhanced S-cone syndrome phenotype only.

**Table 3:** FST threshold comparisons between rod–cone and cone dystrophies.

Parameter	Rod–Cone Dystrophy (*n* = 27)	Cone Dystrophy (*n* = 12)	*p*-Value (Uncorrected)	*p*-Value (Bonferroni) *
Blue FST (dB)	−21.68 ± 13.80	−34.64 ± 17.58	0.0175	0.0700
White FST (dB)	−13.33 ± 6.53	−21.44 ± 13.81	0.0181	0.0722
Red FST (dB)	−20.52 ± 15.28	−31.60 ± 17.38	0.0525	0.2100
Blue–Red Difference (dB)	−8.35 ± 10.37	−11.20 ± 14.60	0.5005	>0.999

Note: After Bonferroni correction for multiple comparisons, no individual FST parameters reach statistical significance (* significance threshold *p* < 0.0125).

**Table 4:** Gene-wise FST comparisons of rod–cone dystrophies.

Gene	*n*	White FST (dB)	Red FST (dB)	Blue FST (dB)	Blue–Red Diff (dB)
*PDE6A*	10	−24.7 ± 18.2	−13.8 ± 7.1	−28.9 ± 16.8	−15.1 ± 14.2
*RHO*	7	−18.9 ± 8.4	−12.7 ± 5.9	−19.8 ± 9.1	−7.1 ± 8.7
*PDE6B*	6	−13.4 ± 4.7	−11.9 ± 6.8	−15.2 ± 7.4	−3.3 ± 5.9
*CNGB1*	4	−16.1 ± 12.8	−18.7 ± 4.2	−17.1 ± 13.7	1.6 ± 11.4

ANOVA *p*-value: 0.231 (white), 0.186 (red), 0.157 (blue), and 0.048 (blue–red diff). Post hoc comparisons with Bonferroni correction showed significant differences between *PDE6A* and *PDE6B* (*p* = 0.02).

**Table 5:** Gene-wise FST comparisons of cone dystrophies.

Gene	*n*	White FST (dB)	Red FST (dB)	Blue FST (dB)	Blue–Red Diff (dB)
*CNGA3*	4	−47.1 ± 3.2	−29.8 ± 11.2	−51.2 ± 10.4	−21.4 ± 7.8
*NR2E3*	4	−13.9 ± 3.1	−12.9 ± 4.2	−19.7 ± 4.8	−6.8 ± 2.9
*GUCY2D*	2	−42.6 ± 1.0	−19.5 ± 3.2	−44.6 ± 7.0	−25.1 ± 3.8
*CNGB3*	2	−48.9 ± 0.1	−30.9 ± 0.5	−56.3 ± 2.4	−25.4 ± 1.9

ANOVA *p*-value: <0.001 (white), 0.002 (red), <0.001 (blue), <0.001 (blue–red diff). Post hoc comparisons with Bonferroni correction showed NR2E3 differed significantly from other cone genes (all *p* < 0.05).

## Data Availability

The original contributions presented in this study are included in the article. Further inquiries can be directed to the corresponding author.

## Abbreviations

The following abbreviations are used in this manuscript:

FST	Full-Field Stimulus Threshold
IRD	Inherited Retinal Dystrophy
ERG	Electroretinography
ISCEV	International Society for Clinical Electrophysiology of Vision
IPS	International Perimetric Society
*CNGA3*	Cyclic Nucleotide-Gated Channel Alpha 3
*CNGB1*	Cyclic Nucleotide-Gated Channel Beta 1
*CNGB3*	Cyclic Nucleotide-Gated Channel Beta 3
*GUCY2D*	Guanylate Cyclase 2D
*NR2E3*	Nuclear Receptor Subfamily 2 Group E Member 3
*PDE6A*	Phosphodiesterase 6A
*PDE6B*	Phosphodiesterase 6B
*RHO*	Rhodopsin
BCVA	Best-Corrected Visual Acuity
OCT	Optical Coherence Tomography
NGS	Next-Generation Sequencing
ACMG	American College of Medical Genetics and Genomics
ROC	Receiver Operating Characteristic
AUC	Area Under the Curve
